# Effects of Chinese cervical fixed-point rotation manipulation on heart rate variability in patients with sympathetic cervical spondylosis based on the theory of biomechanical equilibrium: a study protocol for a randomized controlled trial

**DOI:** 10.3389/fneur.2025.1634946

**Published:** 2025-09-26

**Authors:** Xiayang Zeng, Shengming Zheng, Yunxing Xie, Hongyi Ma, Jinhong Zuo, Zhenyu Fan, Pengfei Qiu

**Affiliations:** ^1^Department of Tui Na, Zhejiang Hospital, Hangzhou, China; ^2^Department of Tui Na, The First Affiliated Hospital of Zhejiang Chinese Medical University (Zhejiang Provincial Hospital of Chinese Medicine), Hangzhou, China; ^3^Department of Acupuncture and Moxibustion, Zhejiang Hospital, Hangzhou, China

**Keywords:** cervical fixed-point rotation manipulation, sympathetic cervical spondylosis, heart rate variability, biomechanical equilibrium, randomized controlled trial

## Abstract

**Background:**

As a complex cervical spondylosis with symptoms involving multiple systems and organs, SCS is easily misdiagnosed and underdiagnosed, and there is a lack of uniform efficacy assessment criteria for SCS, which causes great distress to patients and society. In this study, we will assesse the efficacy of cervical fixed-point rotational manipulation by combining HRV-related parameters and analyzing the correlation between the HRV and SCS.

**Methods/design:**

In this study, 90 participants will be randomly assigned at a 1:1:1 ratio into a test group (cervical fixed-point rotational manipulation manipulation group), a control group (sham manipulation group), or a Western medicine control group, with 30 participants in each group. All participants will be treated for a period of 4 weeks. The primary outcome indicators assessed is the HRV-related parameters, and the secondary outcome indicators are the NDI scale, NPQ scale, and HAMA scale. The assessment time points will be before and after treatment. The follow-up visits will be the first and sixth months after the end of treatment.

**Discussion:**

In this study, SCS are treated with cervical fixed-point rotational manipulation based on the biomechanical balance theory, and HRV-related parameters are used for assessment to analyze the effect of cervical fixed-point rotational manipulation on heart rate variability in patients with SCS.

**Clinical trial registration:**

http://itmctr.ccebtcm.org.cn/, identifier ITMCTR2025000569.

## Background

Sympathetic cervical spondylosis (SCS) refers to the manifestation of cervical spondylosis accompanied by sympathetic symptoms, such as panic and palpitations, chest tightness, dizziness and nausea, in addition to the typical symptoms of cervical spondylosis (1, 2). The manifestation of these symptoms is typically reported by patients diagnosed with cervical spondylosis (CS), and there is considerable heterogeneity in the presentation of symptoms among individuals affected. Furthermore, SCS is subjective in nature, in contrast to CS, which is objective and quantifiable (3). Consequently, the diagnosis and treatment of SCS remain the subject of considerable controversy (4). The nomenclature and medical terminology for SCS are therefore subject to variation in the literature. Some authors refer to it as “posterior cervical sympathetic syndrome” or “Barré-Liéou syndrome” (5), some scholars call it “cervical vertigo” (6) or “cervical angina” (7).

In recent years, there has been an observable acceleration in the rhythm of work and life in general. This phenomenon has coincided with a notable increase in the prevalence of “low-head people,” which has resulted in a corresponding increase in the incidence of cervical spondylosis, particularly among younger people. As with CS, SCS can lead to severe morbidity, but there is frequently an absence of clinician attention, which can be problematic for patients and have a severely deleterious effect on their quality of life (3, 8). Another epidemiological feature of SCS is that it is often not diagnosed or misdiagnosed. The broad spectrum of symptoms, which affect multiple systems and organs, results in patients consulting a wide range of medical specialties, including gastroenterology, neurology, cardiovascular medicine, and otolaryngology, and the absence of a definitive diagnosis often results in inadequate treatment, leading to patient dissatisfaction (9). Clinical studies have shown that approximately 49.1% of patients with cervical spondylosis have depression and anxiety, patients with anxiety and depression have longer-lasting pain and more severe symptoms, and when the treatment effect is not obvious, it further aggravates the patient’s psychological burden, resulting in a vicious cycle of the disease (10, 11). SCS is more complex and is not taken seriously by clinicians; coupled with the lack of obvious treatment effects, patients tend to be more prone to adverse emotional and psychological reactions. The intricacy of the disease’s manifestations profoundly impacts patients’ physical and mental well-being and quality of life, thereby underscoring the imperative for effective and safe therapeutic interventions, which are pivotal health care concerns on a global scale.

The biomechanical equilibrium of the cervical spine is maintained by static and dynamic systems: the static system consists of adjacent vertebrae and their intervertebral discs, joints, ligaments, etc., which play important roles in stabilizing the cervical spine and supporting the skull; the dynamic system is mainly the cervical musculature, collectively referred to as the cervical muscles, which is the primitive driving force of the activities of the cervical spine and is the necessary condition for maintaining the position of the head and neck (12). As a result, maintaining a poor posture for a long period of time leads to neck muscle groups in high-load exercise, excessive muscle tension and stiffness, decreased muscle oxidative metabolism capacity, increased lactic acid levels, pathological changes in the neck muscle groups, disequilibrium of the static system, and the acceleration of soreness and stiffness in the neck muscles (13–15). Spasticity and laxity of tension in the neck muscle groups lead to instability of the intervertebral joints, causing disequilibrium in the dynamic system (13). Therefore, adjusting the muscles and joints, reconstructing the dynamic equilibrium of the cervical spine, and improving the static equilibrium compensation to restore the dynamic equilibrium of the cervical spine can be considered the optimal direction and the best choice for the treatment of SCS at present.

Several studies have shown that physiotherapy and manipulation can alleviate symptoms associated with the sympathetic nervous system (16–18). Chinese spine manipulation (also called “Chinese osteopathy”) is an important part of Chinese massage techniques. The categorization of manipulative techniques is typically dependent on their characteristics and mechanisms of application. Typically, such techniques are divided into three categories: rotation, traction, or a combination of both (19). Cervical rotational manipulation is a prevalent treatment modality for neck pain, with evidence suggesting its efficacy in autonomic function and sensory systems, as well as in pain reduction (20, 21). This approach is endorsed by clinical guidelines (22) and is widely accepted and used as complementary and alternative medicine (23–25). Chinese cervical spine manipulation currently refers to traditional oblique cervical spine manipulation, which involves resetting the entire cervical spine. In fact, oblique cervical spine manipulation has been shown to increase the risk of intervertebral disc and facet joint damage compared with rotational cervical spine manipulation (26). The cervical spine lesions are multivariable and multisegmental, so traditional oblique cervical spine manipulation does not target the lesion segments for treatment. Compared with the conventional cervical oblique wrenching technique, the cervical fixed-point rotation manipulations employed in this study accurately identify diseased cervical segments through a combination of physical examination and imaging techniques. Then, through manipulations, targeted adjustments are made, thereby ensuring a more precise realignment of the biomechanical equilibrium of the cervical spine, both internally and externally.

Heart rate variability (HRV) is a frequently employed metric for assessing autonomic nervous system (ANS) function, and an individual’s responsiveness to HRV-related parameters can often be indicative of their ANS function in maintaining cardiovascular homeostasis (27). The ANS is a neural control system that controls many aspects of respiration, heart rate, and digestion; the sympathetic nervous system (SNS) is a branch of the ANS, and cardiac exogenous sympathetic nerves are mediated through the cervical and cervicothoracic nerves (28–30). Measurements of HRV therefore reflect the activity of the SNS and the parasympathetic nervous system (PNS) (27, 31). Sympathetic cervical spondylosis patients often suffer from symptoms such as chest tightness, panic, and palpitations due to sympathetic nerve stimulation. Currently, an objective and scientific basis for evaluating the efficacy of conservative sympathetic treatment is lacking, and HRV measurements reflect sympathetic nerve activity, which has the advantages of being noninvasive, quantitative, and reproducible; thus, HRV can be used as an indicator for evaluating sympathetic cervical spondylosis. There are more parameters and various measurement indices of the HRV, which include detection indices in the time domain, frequency domain and nonlinear analysis. Among them, time domain and frequency domain analyses are most commonly used. The HRV was generally assessed via the standard deviation of the R–R intervals (SDNN) based on previous studies, high-frequency (HF) values with the root mean square of the neighboring R–R interval differences (RMSSD) and 50 milliseconds as a percentage of the total number of heartbeats (pNN50) as markers of parasympathetic activity (32). HRV time-domain analysis was performed numerically with R-R intervals, and the standard deviation of the mean of the R-R intervals per 5-min segment (SDANN) reflected changes in heart rate, which is a sensitive indicator of sympathetic function but is also influenced by the vagus nerve. Frequency domain analysis is based on low-frequency (LF) and high-frequency (HF) detection, and the ratio of HF/LF reflects the balance between vagal and sympathetic nerves. Time domain analysis can directly reflect the height of the HRV, and a decrease in the HRV suggests an increase in sympathetic nerve tone. Frequency domain analysis reflects the relative tension of the sympathetic and parasympathetic nerves and the balance between them.

We reviewed the literature and reported reports of the use of Chinese medicine and acupuncture to explore the correlation between sympathetic cervical spondylosis and HRV, but no studies have analyzed the correlation between sympathetic cervical spondylosis, HRV and anxiety through Tui Na manipulation in China. We aimed to demonstrate that Tui Na manipulation can improve cardiovascular symptoms in patients with sympathetic cervical spondylosis, explore the correlation between HRV and sympathetic cervical spondylosis, alleviate anxiety, and improve the quality of life. Moreover, we hope these findings will confirm the safety and effectiveness of this therapy and facilitate its clinical promotion.

## Materials and methods

### Participants

#### Baseline assessment

All participants underwent a baseline assessment prior to enrollment, which included demographic information such as sex, age, weight, height, and underlying medical conditions, as well as neck pain-related information such as pain duration, medication use, and other aspects.

#### Recruitment and ethics

Following the acquisition of ethical approval for the clinical trial, SCS participants were recruited from outpatient clinics or wards at Zhejiang Hospital through two methods: offline poster recruitment and online recruitment. At the time of recruitment, participants will be informed of the inclusion criteria, exclusion criteria, trial subgroups, intervention and duration of the intervention in each group, and other important information about the trial. Notably, participants who meet the specified criteria will be furnished with the aforementioned information once more. The informed consent form will be signed by the participants after their consent has been obtained, and they will then be randomly assigned to groups. The clinical trial protocol will adhere to the principles outlined in the Declaration of Helsinki, and its implementation has been sanctioned by the Ethics Committee of Zhejiang Hospital (no. ZJHIRB-2025-026K).

### Diagnostic criteria

Owing to the absence of a universally accepted set of diagnostic criteria for SCS patients within the domain of international medicine, this protocol is to be founded upon the pertinent diagnostic criteria delineated in the 2018 edition of the Chinese Guidelines for Diagnosis, Treatment and Rehabilitation of Cervical Spine Disease, in conjunction with the 10th edition of Tui Na Therapeutics, as stipulated by the Chinese Society of Rehabilitation Medicine:

1. Symptoms: Neck discomfort with sympathetic nerve dysfunction as the main symptom, such as headache, dizziness, tinnitus, and other symptoms of the five senses, with heart rate increasing or slowing down, palpitations and discomfort and other cardiac symptoms.
2. The patient exhibited physical signs indicative of cervical vertebrae pathology, including pressure pain over the front of the transverse processes on both sides of the cervical vertebrae, muscle stiffness, the presence of either striated or rounded nodules, and a positive cervical rotation test.
3. Imaging: Cervical spine X-rays may reveal changes in the cervical curvature, osteophytes, etc. MRI suggests degeneration of the cervical intervertebral discs, and electrocardiography reveals no obvious abnormalities or mild abnormalities.
4. Diseases of systemic organs other than those of ophthalmic, cardiac, cerebral, and auricular origin.

### Inclusion criteria

The participants who meet the following criteria will be included in the study: (1) meetmet the diagnostic criteria of sympathetic cervical spondylosis and exclude other organs and mental diseases; (2) age between 18 and 60 years, and sex will not be limited; (3) currently in the period of sympathetic cervical spondylosis exacerbation, in addition to the main symptom of neck pain, a series of cardiovascular system symptoms such as chest tightness, panic palpitations, dizziness, and sinus rhythm or sinus tachycardia on ordinary electrocardiogram; (4) Positive in head tilt test (HUTT) or neck rotation test; (5) have a Hamilton Anxiety Scale (HAMA) score ≧7, ≦29, in line with mild to moderate anxiety; (6) have clear consciousness, pain perception and discrimination ability, and the ability to clearly describe the condition; and (7) voluntarily accept this clinical programme and sign informed consent.

### Exclusion criteria

The following criteria will be used to exclude patients: (1) those who do not meet the above diagnostic criteria and inclusion criteria; (2) have a combination of heart, brain, liver, kidney, immune system and hematopoietic system and other serious primary diseases and other congenital or acquired diseases that affect the patient’s ability to accurately express his or her feelings and judgment, such as psychosis and dementia; (3) women who are pregnant or intend to become pregnant in the near future and who are breastfeeding; (4) patients with vertebral fracture, vertebral deformity, tuberculosis in the spinal canal, or concomitant spinal cord cervical spondylosis or vertebral artery cervical spondylosis; (5) those with severe skin ulceration of the neck skin; and (6) those with poor compliance and who do not cooperate with the treatment program.

### Randomization and allocation concealment

The randomization process will be conducted by an independent statistician who is not involved in the trial. The statistician will use the statistical software SPSS 20.0 to allocate 90 serial numbers at a ratio of 1:1:1 and 30 numbers for each of the three groups of the trial, each randomly corresponding to the corresponding subgroup, and subsequently record the random number and its corresponding subgroup on the computer. The envelopes containing the serial numbers will be placed in opaque, sealed cardboard boxes, which will be handed over to an independent person for safekeeping to ensure the confidentiality and security of the grouping information. Participants who meet the screening criteria will be randomly selected the envelopes containing serial numbers from cardboard boxes and then filled in the case report form. The envelopes will be opened to check the serial numbers, and the statistician will look up the grouping corresponding to the serial numbers on the computer to inform the operator and record the grouping, which will not subject to modification.

### Blinding

Prior to the start of the study, personnel will be trained in the segregation of duties to ensure blinding. The trial will be blinded to some participants and assessors; except for those in the Western medicine group, who will aware of their grouping and need to take medication, other participants in both groups will unaware of their grouping, they arel only be told that they will accept manipulative treatment and that they will have a 50% chance of being placed in the test group (cervical fixed-point rotation manipulation group) or the control group (pseudomanipulative therapy group). The statistical results will be analysed by an independent personnel who will unaware of the grouping, to ensure the implementation of the blind method. At the same time, statisticians will calculate the Bang Blinding Index. Although manipulative therapists are aware of the grouping of participants, they will not involved in the evaluation and analysis of the results of the trial.

The implementation of the blind method in this trial was not comprehensive and could not achieve complete blinding. Patients in the Western medicine group knew what treatment they were receiving, so the results may be biased. We will clearly indicate the possible sources of bias in the blind method, the assessment methods, and the extent of their impact on the results in the clinical research report. We will objectively discuss the impact of bias on the strength and credibility of the research conclusions, as well as the scope of application and promotion of the research results. Additionally, we will administer a questionnaire to patients after treatment to assess their treatment expectations, thereby quantifying the success of the blinded design implementation.

### Trial design

The main objective of this study is to observe the effect of cervical localized rotation manipulations on heart rate variability in patients with SCS. Through 24-h ambulatory electrocardiogram, changes in HRV during the treatment of sympathetic cervical spondylopathy can be observed more intuitively and objectively. To evaluate the efficacy of cervical fixed-point rotation manipulations more objectively, a pseudomanipulative therapy group will be added to the test group for the control group, and it will be observed whether there is a difference in efficacy between cervical fixed-point rotation manipulations and pseudomanipulative therapy to further clarify the efficacy of cervical fixed-point rotation manipulations.

**Figure 1:**
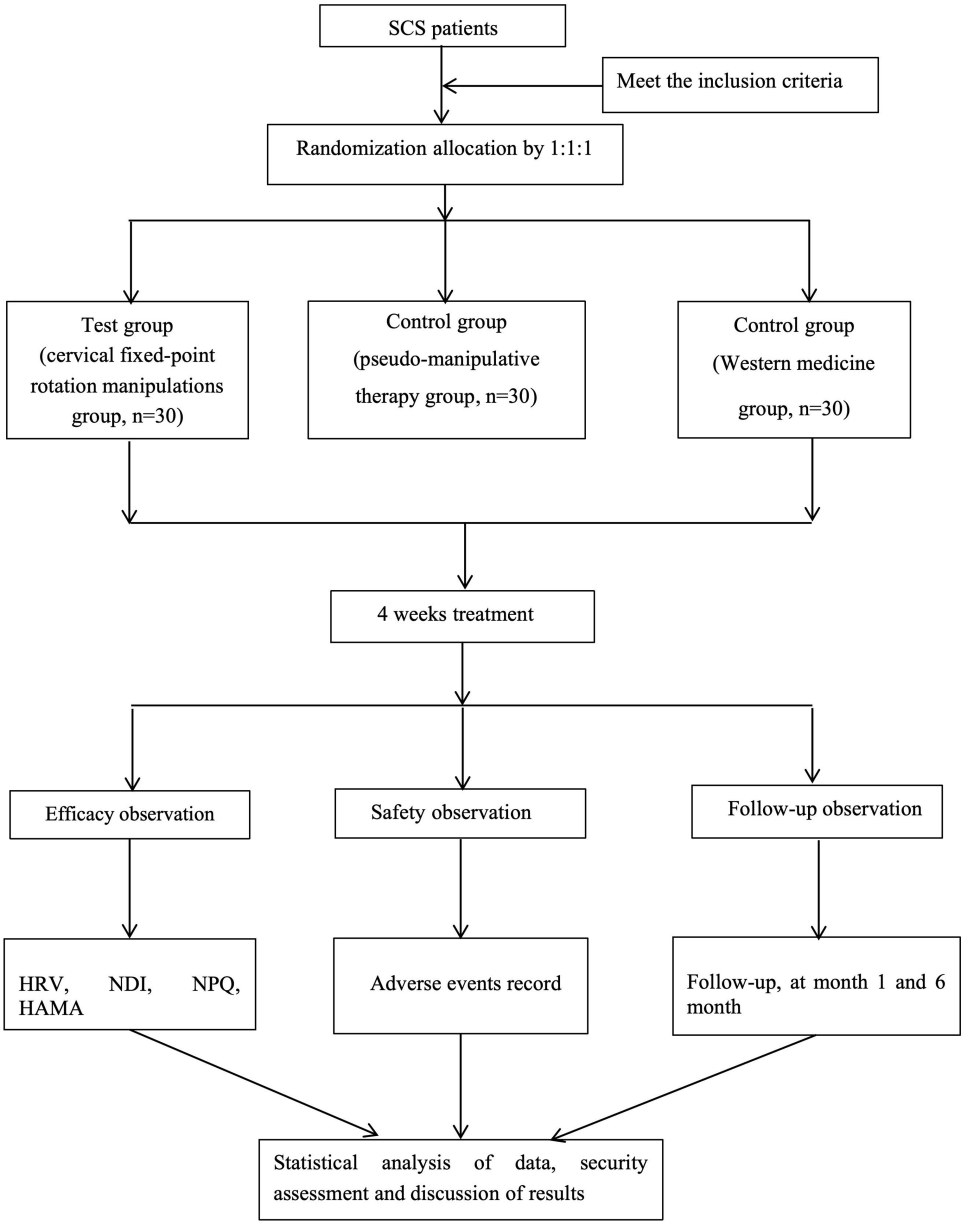
Flow chart of the study process. SCS, Sympathetic Cervical Spondylosis; HRV, Heart Rate Variability; NDI, Cervical Dysfunction Index; NPQ, Neck Pain Questionnaire; HAMA, Hamilton Anxiety Scale.

**Figure 2:**
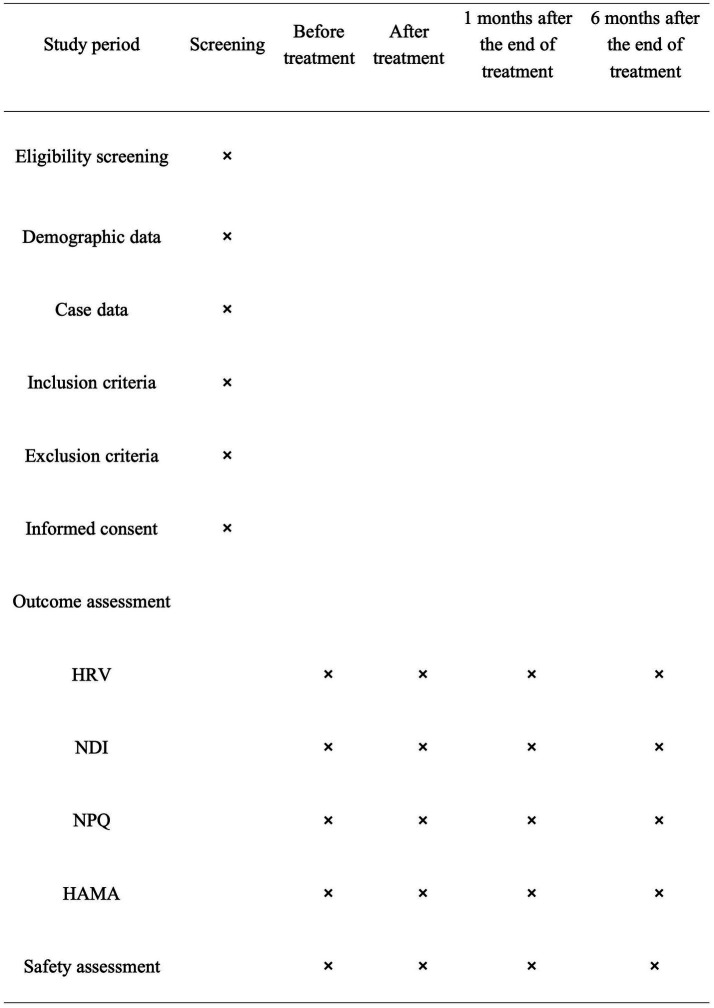
Schedule of enrolment, treatments, and assessments. ×, required; HRV, Heart Rate Variability; NDI, Cervical Dysfunction Index; NPQ, Neck Pain Questionnaire; HAMA, Hamilton Anxiety Scale.

This study is a randomized controlled trial conducted at Zhejiang Hospital to assess the effect of cervical fixed-point rotation manipulations on heart rate variability in patients with SCS. A total of 90 participants with SCS who meet the criteria will be randomly assigned to the test group (cervical fixed-point rotation manipulation group), control group (pseudomanipulative therapy group), or Western medicine group. The study has been registered on the International Traditional Medicine Clinical Trial Registry, which is accredited by the World Health Organization as a Level 1 registry, at the following URL: http://itmctr.ccebtcm.org.cn/ (the Clinical trail number: NO. ITMCTR2025000569). The protocol reporting will adhere rigorously to the SPIRIT guidelines (33). The process of the study is illustrated in Figure 1, which presents a flowchart. Furthermore, the schedule for the enrollment, treatment and assessment of the trial is show in Figure 2.

### Intervention

#### Test group (cervical fixed-point rotation manipulation group)

1. Muscle relaxation: the participant will assume the prone position or sitting position; the practitioner will relaxe the muscles with traditional Tui Na techniques; and the doctor will relaxe the muscles of the neck, shoulder, and upper back with kneading, plucking, and pinching. This method focuses on pressing and kneading at the strips or hard knots with points, plucking, and generally finds pressure points or hard knots in the trapezius, sternocleidomastoid and transverse cervical vertebrae, and kneading and kneading techniques are performed for approximately 10–15 min. Once the muscles are relaxed, the following points will be massaged and stimulated with the thumb or elbow joint: Fengfu (DU16), Fengchi (Gb20), Yamen (DU15), Jiaji points of cervical (EX-B2), Dazhui (DU14), Jingbailao (EX-HN14), Tianzong (SI11), Neiguan (PC6), Waiguan (SJ5), Hegu (LI4), Ashi point. Table 1 shows the location of the acupuncture points.
2. Cervical localized rotation manipulations: Rotation manipulations will be applied precisely to the diseased cervical vertebral segment according to the specifics of the examination and imaging; for example, the C2 spinous process deviates to the right side, or the small joints are misaligned. After muscle relaxation, the patient will assume a sitting position, and the physician will stand behind the patient’s right side, with the right thumb against the patient’s C2 spinous process and the remaining four fingers placed on the contralateral side of the lower neck to help. The participant’s head will be bowed with the lower jaw placed in the practitioner’s right elbow socket, the face will be tilted to the right, and the practitioner will hold the participant’s left mandibular angle with the lower arm of the right hand to assist, the practitioner’s right arm will be the participant’s head upward support, the participants will be exert upward pulling and stretching force, and then the participant’s head will be placed to the right side lateral flexion to the maximum limit and then slightly turned to the left side, and then the head right lateral flexion movement will be suddenly increased to an amplitude of approximately 3–5°. The left thumb simultaneously will push the cervical spinous process to the left, at which point a popping sound can be heard. At the end of the manipulation, whether the cervical spinous process distortion is corrected; if the distortion is still present, the above maneuver will be repeated 2–3 times. The course of treatment will be as follows: once every other day, three times a week, four consecutive weeks of treatment, for a total of 12 times. Follow-ups will be conducted 1 month and 6 months after the end of treatment.

**Table 1:** Location of acupoints for treating SCS.

Acupoints	Location
Fengfu (DU16)	In the neck, 1 cun straight up from the middle of the posterior hairline, straight down from the occipital convexity, and in the depression between the trapezius muscles on both sides
Fengchi (Gb20)	In the neck, when below the occipital bone, in the depression between the sternocleidomastoid muscle and the upper end of the trapezius muscle.
Yamen (DU15)	In the neck, 0.5 cun straight up from the middle of the back of the hairline, under the 1st cervical vertebra.
Jiaji points of cervical (EX-B2)	The first cervical vertebra to the seventh lumbar vertebra, 0.5 cun below the spinous process of each vertebra
Dazhui (DU14)	In the inferior recess of the spinous process of the 7th cervical vertebra.
Jingbailao (EX-HN14)	Extra-meridian acupuncture point, located at the neck, 2 inches above the depression under the spinous process of the 7th cervical vertebra (the point of the great vertebrae), 1 cun away from the posterior midline
Tianzong (SI11)	Located in the scapular region, in the depression at the intersection of the upper 1/3 and lower 2/3 of the line connecting the midpoint of the scapular ridge and the lower angle of the scapula, in the infraspinatus muscle in the centre of the infraspinatus fossa
Neiguan (PC6)	Located on the palmar side of the forearm, 2 cun above the transverse carpal stripe, between the tendon of the palmaris longus and the flexor carpi radialis tendon
Waiguan (SJ5)	Located on the dorsal side of the forearm, in the posterior region of the forearm, 2 cun above the transverse stripe on the dorsal distal side of the wrist, and at the midpoint of the interspace between the ulna and radius.
Hegu (LI4)	On the back of the hand, between the 1st and 2nd metacarpals, when the midpoint of the radial side of the second metacarpal.
Ashi points	The point that feels most painful to localized pressure

#### Control group (pomanipulative therapy group)

Pseudomanipulative therapy involves light touching on the patient’s neck muscles, starting with kneading to relax the muscles, moving the hand every 5 s to prevent the patient’s body from reacting mechanically to prolonged force or touch, and relaxing the hand during treatment to reduce the concentration of stress without pressing too much on pressure points or muscle striae nodules. During the manipulation process, the pressure applied should not be too heavy; gentle pressure should be used, with each muscle being pressed for no more than 5 s. When performing cervical fixed-point rotation manipulation, it is not necessary to locate the affected cervical spine segment. Under the premise of ensuring safety, there is no requirement for the angle, force, or accuracy of the maneuver, and the joint popping sound produced by the trigger is not pursued. To ensure the implementation of the patient’s blind method, the two treatment implementers will be required to maintain a similar maneuver to ensure the same contact time. It is guaranteed that the subjects will not contact with the manipulation operator outside of the treatment session. The course of treatment will be as follows: once every other day, three times a week, four consecutive weeks of treatment, for a total of 12 times. Follow-ups will be conducted 1 month and 6 months after the end of treatment.

#### Western medicine group

Mecobalamin (Eisai China Pharmaceutical Co., Ltd., 500 μg/tablet) 500 μg/dose, 3 times a day, supplemented with celecoxib capsules (Pfizer Pharmaceutical Co. Ltd., 0.2 g/capsule) 0.2 g/d, 1 time/d, for pain relief in those with obvious pain, for a duration of 4 weeks. Follow-ups will be conducted one month and six months after the end of treatment.

### Outcome measures

#### Primary outcome

HRV-Related Parameters: This study will utilise a 24-h ambulatory electrocardiograph to measure three distinct groups of HRV parameters, both prior to and following treatment. The primary parameters will be evaluated encompassed the standard deviation of the mean of the R-R intervals per 5-min segment (SDANN), the standard deviation of the R-R intervals (SDNN), the proportion of neighboring R-R interval differences greater than 50 milliseconds as a percentage of the total number of heartbeats (PNN50), and the root mean square of the neighboring R-R interval differences (rMMSD).

#### Secondary outcome

1. Cervical Dysfunction Index (NDI) and Neck Pain Questionnaire (NPQ): participants will be assessed for cervical spine function and pain via a questionnaire. The NDI is a significant instrument for the evaluation of cervical spine function. The NDI is categorized into four levels: 0–20% for mild dysfunction, 21–40% for moderate dysfunction, 41–60% for severe dysfunction, 61–80% for very severe dysfunction, and 90–100% for complete dysfunction. The NPQ Neck Pain Scale, on the other hand, uses a 9-point scale ranging from 0 to 100, with higher scores denoting more severe neck pain and conditions. An elevated score on the scale is therefore indicative of a more severe condition.
2. Hamilton anxiety scale (HAMA): a questionnaire will be used to rate the patients’ anxiety symptoms. A total of 14 areas of questions are included to classify the anxiety factors into two categories: somatic and psychogenic. The main items are seven to thirteen for somatic anxiety and one to six and fourteen for psychogenic anxiety. All items of the HAMA are rated on a 5-point scale from 0–4, with the criteria for each level being (0) asymptomatic, (1) mild, (2) moderate, (3) severe, and (4) extremely severe, with a total of fourteen items to record a total score, with higher scores representing a more anxious state.

#### Follow-up observation

Patients will be monitored posttreatment, with follow-up assessments conducted at one month and six months. These assessments will be conducted either in person at the hospital or via telephone, and home visits will be arranged as necessary. The primary method of data collection involve the administration of questionnaires. For those demonstrating substantial improvement in panic and palpitations and significant changes in heart rate variability, an ambulatory electrocardiogram will be conducted at the time of follow-up to assess the long-term effects of manipulative therapy.

#### Safety assessment and adverse events

During the study period, the researchers will record the adverse events at any time and assess whether they are related to the intervention. If they are related to the intervention, the intervention will be stopped immediately and recorded in the CRF form and report to the Ethics Committee of Zhejiang Hospital, and the participants will be given appropriate financial compensation. Adverse events occurring in the different treatment regimens in this study will statistically compared to evaluate the safety of the different treatment regimens. The safety evaluation is graded as follows:

Grade 1: safe, without any adverse reactions; no abnormality in the safety index examination.
Grade 2: relatively safe, with mild adverse reactions, can continue the treatment without any treatment; no abnormality in the safety index examination.
Grade 3: safety concerns, moderate adverse reactions, and treatment can be continued; safety indices are mildly abnormal.
Grade 4: Discontinue the study due to serious adverse reactions or if there is an obvious abnormality in the safety index examination.

#### Data collection and management

The data of the included participants in this study will all be recorded on CRFs, and the clinical trial data will be recorded by an independent third person on a uniformly printed CRF sheet. Excel software will be used to create a database, with data entered independently by two people. All physical CRF texts will be kept in a locked cabinet in the researcher’s office. The Excel database and CRF texts will be authorized only to the data manager and statistician to ensure data security and protection of participant privacy.

#### Statistical methods

According to the prestudy observations, the best effective rate of the test group was 85%, the effective rate of the pseudomanipulative therapy control group was 40%, and the effective rate of the Western medicine group was 47%. The sample size estimation formula for the comparison of multiple sample rates was used to calculate P_max_ = 0.85, P_min_ = 0.40, *α* = 0.05, *β* = 0.10, and *ν* = 3–1 = 2, and the checking of the attached table yielded *λ* = 12.65. The number of sample cases was distributed between the groups according to a ratio of 1:1:1, and a unilateral test was used. *N* = 27 and considering a 10% shedding rate, 30 samples were needed in each group, for a total of 90 in the three groups.


$$n=\frac{2\lambda }{{\left(2\sin^{-1}\sqrt{P_{\max}}-2\sin^{-1}\sqrt{P_{\min}}\right)}^{2}}$$

$$=\frac{2\times 12.65}{{\left(\sin^{-1}\sqrt{0.85}-\sin^{-1}\sqrt{0.40}\right)}^{2}}$$

=26.6,take27

#### Statistical analysis

The data obtained in this study will be entered into the Excel database by two independent persons, and the data will be analyzed by statisticians through SPSS (Statistical Product and Service Solutions) 20.0 statistical analysis software. Count data will be analyzed via the X2 test and the rank sum test for rank data. Measurements will be first tested for normality via the Shapiro–Wilk test, and measurements that conformed to a normal distribution were described via the mean plus minus standard deviation (X ± S). Comparisons between pre- and posttreatment values within groups will be made via the paired-samples t test, and comparisons before and after treatment between groups will be made using the performed via one-way analysis of variance (ANOVA). The results will be analyzed, with *p* < 0.05 as the judgment criterion for significant differences. The rank sum test will be used for nonconformity to a normal distribution.

## Discussion

Symptoms such as palpitations and panic in SCS patients have been found to be the result of stimulation of sympathetic nerves in the posterior longitudinal ligament and intervertebral plexus (34, 35). Several studies have shown that the posterior longitudinal ligament is rich in sensory nerve fibers and is connected to the sympathetic nerves by synapses (18, 36, 37). When these sensory nerve fibers are stimulated, the excitability of the sympathetic fibers is enhanced (38, 39). The stimulation of sympathetic nerves has been demonstrated to result in the release of monoamine transmitters, such as NPY. These neurotransmitters modulate blood vessels and induce the release of substance *P* from sensory nerves, which in turn promotes the dilation of large arteries and further enhances stimulation (38, 40). As a consequence of these stimuli, the sympathetic nerves may trigger the relevant organ systems, thus engendering sympathetic symptoms, including dizziness, palpitations, and tinnitus (41). Consequently, the cervical sympathetic nerves exert a broad regulatory influence on the visceral organs (42). Given the intricacies inherent to SCS, coupled with the dearth of an unequivocal pharmacological resolution, massage therapy, an integral component of complementary and alternative medicine, is garnering heightened interest from those afflicted by SCS. This predilection stems from massage therapy’s safety profile, affordability, and accessibility, making it a viable therapeutic alternative.

Among the numerous cervical massage techniques, cervical rotational manipulation has garnered favorable feedback in the management of cervical spondylosis (20). As Moser et al. reported, cervical rotation manipulation has been demonstrated to reduce reverse blood flow and velocity within the vertebral artery (43). Guan et al. conducted a series of experiments on animals to ascertain the safety of cervical rotation manipulation (44). The results of these experiments demonstrated that this manipulation technique does not result in damage to blood cells, thus confirming its safety (44). With the application of biological finite element technology in the field of biomechanics, several scholars have found through computational modeling that cervical rotation manipulation changes the stress distribution around the cervical spine and improves the biomechanical equilibrium around the cervical spine (21).

Compared to other traditional cervical spine manipulation techniques, the cervical fixed-point rotation manipulation is highly targeted during operation, with a key feature being its “accuracy.” First, precise localization is essential. During this technique, the practitioner first performs a palpation to determine whether the pathological site is in the muscles (muscle bands) or the cervical spine (joint dislocation), and then confirms the dislocated joint based on imaging diagnostics. Secondly, the timing of the manipulation is precise. During the procedure, the cervical spine should be rotated to its maximum extent or moved to the “trigger point” where resistance is felt, at which point a controlled, slightly increased-amplitude, instantaneous manipulation is performed. Compared to traditional cervical rotation manipulation techniques, the cervical fixed-point rotation manipulation technique is more targeted and precise in addressing the affected cervical segments and muscles, has the effect of correcting joint dislocation, realigning anatomical structures, and relieving muscle spasms, resulting in clearer effects. Clinically, it is primarily used for cervical spondylosis with nerve compression, such as radicular cervical spondylosis and sympathetic cervical spondylosis.

Previous studies of cervical rotational manipulation did not explore the patient’s cervical spine lesion nodes for treatment and consider other systemic diseases, did not truly improve the patient’s symptoms, and mostly assessed the efficacy in the form of questionnaires, which lacked objective and scientific evaluation standards; however, HRV has the advantages of being accurate, noninvasive, simple to operate, and dynamically observable and can be used as an objective standard for evaluating the efficacy of treating patients with SCS. This study is the first to analyze the correlation between heart rate variability and the SCS by means of Tui Na manipulation and to propose an objective and reliable evaluation index.

Despite the proven effectiveness of cervical rotational manipulation in the treatment of cervical spondylosis, its safety remains a concern. Many people are concerned that improper manipulation may cause more damage to the blood vessels and nerves in the neck. Because of the requirements for the angle and strength of the manipulation, more clinical protocols are needed to ensure the safety of the manipulation and to better serve the clinic. In this study, for the first time, the cervical spine was adjusted segmentally and locally through cervical fixed-point rotational manipulation, which was more targeted to the cervical spine of the diseased segments, and manipulative operations and strengths were described during the study to ensure that the manipulative operations were carried out within the safety limits.

However, there are still limitations in this study. Although HRV can be used as an objective criterion for assessing the efficacy of SCS patients, there are episodes and flattening of symptoms in SCS patients, and the assessment is more accurate only when the patient is in the episodic phase with symptoms such as panic attacks and palpitations, so there is a limitation on the time point of assessment. In addition, there are few clinical reports on the correlation between SCS symptoms and HRV, and the reliability of HRV assessment is controversial. Therefore, more attention should be given to the validity and reliability of the HRV in the assessment of efficacy in SCS patients in future clinical studies. Greater emphasis must be placed on the necessity of standardizing and assessing the safety of cervical rotation manipulation. In the future, the employment of biological finite element analysis will facilitate the calculation of more precise manipulation angles and force parameters; this, in turn, should result in a more effective promotion of the cervical method of manipulation, leading to substantial improvements in the quality of life of SCS patients and, by extension, the overall health of the population.

## Conclusion

The aim of the present study was to assess the clinical efficacy of cervical fixed-point rotational manipulation in the treatment of SCS using HRV-related parameters, the NDI scale, the NPQ scale and the HAMA scale. Furthermore, this study analyzed the correlation between heart rate variability and the SCS. The results of this study will be used to determine the efficacy of cervical fixed-point rotational manipulation for the treatment of SCS and to examine whether HRV can be used as an evidence-based method for assessing SCS. These results may change the approach to conservative treatment for patients with SCS and enhance patient quality of life.
